# Telephone care coordination for smokers in VA mental health clinics: protocol for a hybrid type-2 effectiveness-implementation trial

**DOI:** 10.1186/1940-0640-8-7

**Published:** 2013-03-15

**Authors:** Erin Rogers, Senaida Fernandez, Colleen Gillespie, David Smelson, Hildi J Hagedorn, Brian Elbel, David Kalman, Alfredo Axtmayer, Karishma Kurowski, Scott E Sherman

**Affiliations:** 1 VA New York Harbor Healthcare System, 423 East 23rd Street – 15N, New York, NY 10010, USA; 2 Department of Medicine, Division of General Internal Medicine, New York University School of Medicine, 550 First Avenue, New York, NY 10016, USA; 3 Department of Population Health, New York University School of Medicine, 550 First Avenue, New York, NY 10016, USA; 4 Edith Nourse Rogers Memorial Veterans Hospital, 200 Springs Road, Bedford, MA 01730, USA; 5 University of Massachusetts Medical School, 365 Plantation Street, Worcester, MA 01605, USA; 6 Minneapolis VA Medical Center, 1 Veterans Dr, Minneapolis, MN 55417, USA; 7 Department of Psychiatry, University of Minnesota, School of Medicine, 2450 Riverside Avenue, South Minneapolis, MN 55454, USA; 8 California Breast Cancer Research Program University of California Office of the President, 300 Lakeside Drive, 6th Floor, Oakland, CA 94612-3550, USA

**Keywords:** Tobacco, Smoking, Mental health, Intervention, Implementation, Psychiatry

## Abstract

**Background:**

This paper describes an innovative protocol for a type-II hybrid effectiveness-implementation trial that is evaluating a smoking cessation telephone care coordination program for Veterans Health Administration (VA) mental-health clinic patients. As a hybrid trial, the protocol combines implementation science and clinical trial methods and outcomes that can inform future cessation studies and the implementation of tobacco cessation programs into routine care. The primary objectives of the trial are (1) to evaluate the process of adapting, implementing, and sustaining a smoking cessation telephone care coordination program in VA mental health clinics, (2) to determine the effectiveness of the program in promoting long-term abstinence from smoking among mental health patients, and (3) to compare the effectiveness of telephone counseling delivered by VA staff with that delivered by state quitlines.

**Methods/design:**

The care coordination program is being implemented at six VA facilities. VA mental health providers refer patients to the program via an electronic medical record consult. Program staff call referred patients to offer enrollment. All patients who enroll receive a self-help booklet, mailed smoking cessation medications, and proactive multi-call telephone counseling. Participants are randomized to receive this counseling from VA staff or their state’s quitline. Four primary implementation strategies are being used to optimize program implementation and sustainability: blended facilitation, provider training, informatics support, and provider feedback. A three-phase formative evaluation is being conducted to identify barriers to, and facilitators for, program implementation and sustainability. A mixed-methods approach is being used to collect quantitative clinical effectiveness data (e.g., self-reported abstinence at six months) and both quantitative and qualitative implementation data (e.g., provider referral rates, coded interviews with providers). Summative data will be analyzed using the Reach Effectiveness Adoption Implementation Maintenance (RE-AIM) framework.

**Discussion:**

This paper describes the rationale and methods of a trial designed to simultaneously study the clinical effectiveness and implementation of a telephone smoking cessation program for smokers using VA mental health clinics. Such hybrid designs are an important methodological design that can shorten the time between the development of an intervention and its translation into routine clinical care.

**Trial registration:**

ClinicalTrials.gov NCT00724308

## Background

Smoking is responsible for 435,000 deaths per year [1]. Persons with a mental health diagnosis have particularly high rates of tobacco use and consume over 46% of cigarettes sold in the US [2,3]. Patients with bipolar disorder or schizophrenia have the highest smoking rates (69% and 58-90%, respectively) followed by those with post-traumatic stress disorder (45-63%) and depression (31-51%) [3-5]. Nicotine replacement therapy (NRT), bupropion, and behavioral interventions are recognized as effective smoking interventions for patients with and without mental illness [6-10]. Although in-person behavioral counseling is recognized as the most effective form of behavioral intervention for smoking, telephone-based counseling for smoking cessation has greater reach than in-person counseling and is easily amenable to tailoring based on patient needs [9,11-13].

Every US state, as well as Washington DC, has a telephone “quitline,” which residents can call to receive self-help materials, counseling, and, in some states, free or discounted medications. Unfortunately, telephone counseling has been underutilized among smokers with mental health diagnoses, and there are no published studies examining its effectiveness specifically in a mental health sample despite evidence that persons with a mental illness accept, participate in, and benefit from telephone counseling programs for other purposes [14-16]. A single trial of telephone care coordination for smokers in primary care clinics that connected patients to their state quitline for counseling included patients with mental health diagnoses and showed that the program increased long-term abstinence compared with usual care [17]. However, the number of mental health patients included in the trial was too small to make conclusions about the program’s effectiveness among mental health patients, and it is unknown whether the program would function similarly outside the primary care setting. In addition, it is unclear whether state quitlines can adequately serve smokers with mental illness, who may need more intensive treatment than the general population, especially those quitlines that offer only a single call or a small number of calls per client. A 2007 survey of state quitlines revealed that none of the US quitlines had specialized services for clients with mental illness, and only 6% had self-help materials tailored for smokers with mental health conditions [18]. Therefore, mental health patients may require a larger number of calls and more tailoring than is currently available from the majority of state quitlines.

We are conducting a hybrid effectiveness-implementation trial [19] to simultaneously study the clinical effectiveness and implementation of a telephone care coordination program for smokers using Veterans Health Administration (VA) mental health clinics. Hybrid effectiveness-implementation study designs are an important and innovative methodological advance designed to shorten the time between the development of an intervention and its translation into routine clinical care. According to Curran et al. [19], there are three primary types of hybrid designs that lie on a continuum from clinical effectiveness research to implementation research. Type I designs focus on studying the clinical effectiveness of an intervention while observing and gathering information on the implementation of that intervention into routine care. Type II designs prioritize clinical and implementation outcomes equally by concurrently studying the clinical effectiveness of an intervention and one or more strategies for implementing that intervention. Type III designs focus on implementation outcomes by studying one or more implementation strategies for an intervention while gathering information about the intervention’s clinical outcomes. Our trial represents a Type II hybrid design. The trial will estimate the clinical effectiveness of the care coordination program with VA mental health patients, compare the effectiveness of quitline counseling to counseling from VA staff trained to work with smokers with mental illness, identify important barriers of and facilitators for implementing the program in VA mental health treatment settings, and describe key outcomes of four non-randomized implementation strategies. The trial has three primary objectives: (1) evaluate the process of adapting, implementing, and sustaining a smoking cessation telephone care coordination program in VA mental health clinics; (2) determine the effectiveness of the program in promoting long-term abstinence from smoking among VA mental health clinic patients; and (3) compare the effectiveness of telephone counseling delivered by VA staff trained to work with smokers who have a mental health condition with that delivered by state quitlines. This paper describes the design and methodology of the trial.

## Methods/design

### Overview

**Figure 1:**
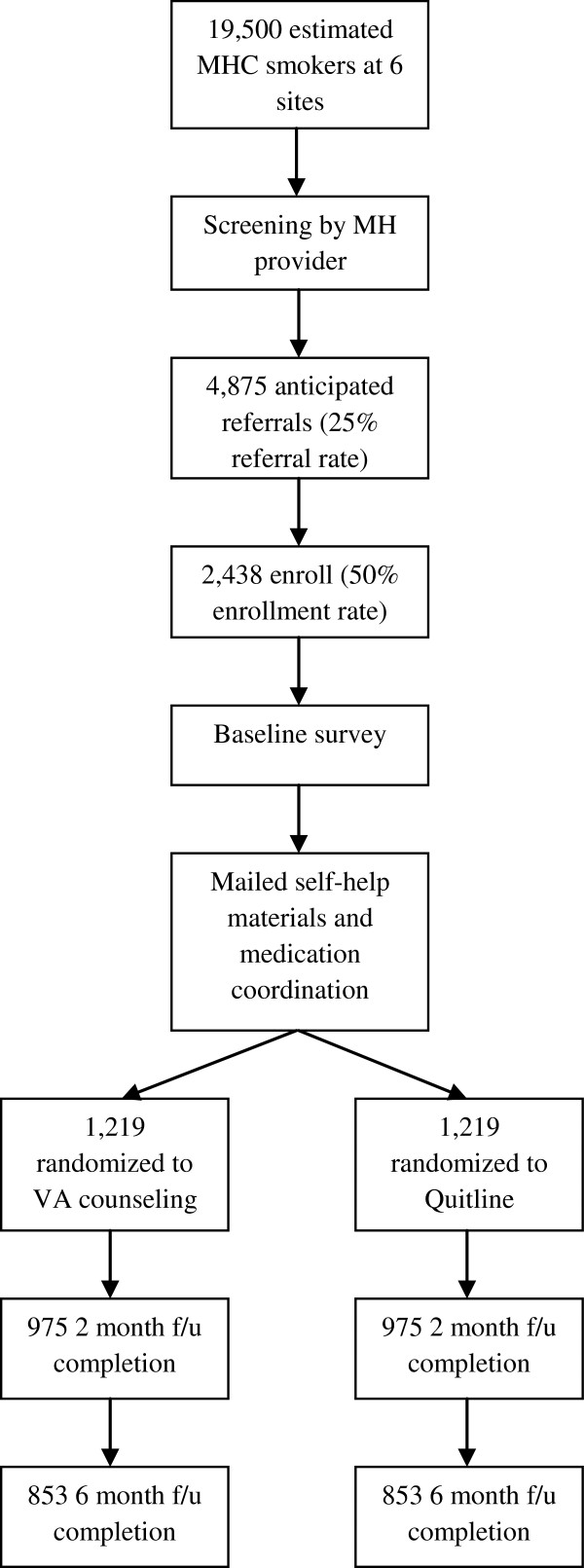
CONSORT flow diagram.

Figure 1 provides an overview of the clinical effectiveness portion of the trial’s design. We are implementing a telephone care coordination program (called the Telephone Quality Improvement Trial in Mental Health, or “TeleQuitMH”) at six VA facilities. Mental health providers refer their smoking patients to the program via electronic medical record (EMR) consult. The program calls referred patients to offer enrollment using the patient’s phone numbers listed in the EMR. Enrolled patients receive smoking cessation medications and multi-session, proactive telephone counseling. In a one-to-one fashion, we randomize patients to receive the proactive telephone counseling from a VA counselor or their state quitline. We survey patients by phone at baseline, two months, and six months to assess outcomes. We are using four organizational- or provider-level interventions to optimize program implementation: blended facilitation, provider training, informatics support, and provider feedback. We are conducting a three-phase formative evaluation to identify barriers to and facilitators for program implementation.

### Study sites

Table 1 describes the structure and target patient population of the six participating facilities in New York, New Jersey, Massachusetts, Vermont, New Hampshire, and Rhode Island. Each site includes one or more medical centers and multiple outpatient clinics. Like all VA sites, our participating facilities use a common EMR that includes clinical reminders for tobacco use screening and treatment. Usual care for tobacco use at our sites includes patient access to NRT, Bupropion, and Varenicline through regular prescribing providers and in-person smoking cessation clinics offering group counseling.

**Table 1:** Overview of participating facilities

**Facility name**	**States served**	**Estimated number of MHC smokers**	**% Female**	**Number of affiliated medical centers**	**Number of affiliated CBOCs**	**R&D service**
New York Harbor HCS	NY, NJ	4,500	8	2	6	Y
Bronx VAMC	NY, NJ	3,900	6	1	4	Y
New Jersey HCS	NJ	5,000	7	2	10	Y
Bedford VAMC	MA	2,300	5	1	5	Y
White River Junction VAMC	VT, NH	1,800	9	1	6	Y
Providence VAMC	RI	2,000	6	1	5	Y

*HCS* = Healthcare System; *VAMC* = VA Medical Center; *CBOC* = Community-Based Outpatient Clinic; *MHC* = Mental Health Clinic.

### Participants

All smokers who see a VA mental health provider at participating sites during our intervention period can be referred to TeleQuitMH. The decision to refer a patient is based on the provider’s assessment of the appropriateness of referral to a telephone program and the patient’s agreement to the referral. Eligibility criteria for study participation include referral from a VA mental health provider, access to a telephone, and access to a mailing address (a post-office box is acceptable). An estimated 19,500 smokers use VA mental health services annually at our six sites. Based on a prior study testing a similar smoking care coordination program in VA primary care [17], we anticipate a 25% referral rate and a 50% enrollment rate among the referred, giving a total of about 2400 patients enrolling in the program during our intervention period (1200 of whom will be randomized to VA counseling and 1200 to quitline counseling).

### Clinical intervention: telephone care-coordination program

The TeleQuitMH care coordination program consists of the following sequential elements. The program begins with a provider-level intervention that enables the referral of patients to tobacco cessation treatment. After the referral, patient-level treatment intervention begins.

1. ***Simple EMR referral****—*The care coordination program takes advantage of VA usual care that requires providers to screen patients for tobacco use and either provide them with counseling or refer them to treatment during routine visits. We are not attempting to standardize the screening process or counseling given by mental health providers. Our study introduces a new treatment referral option for providers—that of referring patients to TeleQuitMH via a new electronic consult in the EMR. Providers have the option to confirm the patient’s contact information and enter free-text information about their patient in the consult screen, but these responses are not required. Providers can access the consult either through the consult menu or through the tobacco use clinical reminder screens.

2. ***Proactive outreach***—Once our program receives a referral, we mail the patient a welcome packet that includes information about the TeleQuitMH program and study and a brochure containing all elements of informed consult. One week following this mailing, program staff call the patient using the phone number in his or her medical record. Staff make five attempts over two weeks to reach referred patients by phone to discuss the smoking cessation program, offer enrollment, and complete the verbal informed-consent process. Patients who enroll in treatment are provided with the next four elements of the program described below. Patients not interested in treatment have the opportunity to enroll in the follow-up surveys only.

3. ***Medication coordination***—All enrolled patients can receive mailed smoking cessation medications unless they decline or have contraindications noted by their referring provider. The method with which the TeleQuitMH program provides medications varies by site and is determined by a site Clinical Advisory Committee formed before site implementation. This committee consists of key stakeholders from the departments of Psychiatry, Social Work, Psychology, Pharmacy, and Smoking Cessation. There are three prescribing methods from which the Clinical Advisory Committee can choose:

a. The site can designate one or two prescribers who will complete all prescriptions for participants at their site. Based on the success of the primary care-based projects that used a designated provider, we recommend this method to all sites.

b. Referring prescribers prescribe for their patients at the time of referral to TeleQuitMH. If a patient was referred by a nonprescriber (e.g., a social worker), or a referring prescriber did not prescribe at the time of referral, a TeleQuitMH research assistant contacts the patient’s regular psychiatrist or primary care provider to request medications.

c. A combination of methods 1 and 2 is used. Referring prescribers can prescribe for their patients at the time of referral. If a patient was referred by a nonprescriber or the referring prescriber did not prescribe at the time of referral, a TeleQuitMH assistant will contact a site-designated prescriber to request medications.

4. ***Self****-****help materials***—All enrolled patients receive a mailed self-help booklet developed jointly by the University of Colorado School of Medicine and Colorado Department of Public Health and Environment. The two-page booklet provides brief advice on preparing to quit smoking, setting a quit date, and preventing relapse. The booklet also provides corrective information on five common myths about smoking among persons with a mental health diagnosis. We tailored this booklet for a VA mental health population by including brief advice on linking smoking cessation treatment with regular mental health care and changing the booklet’s imagery to include pictures of persons in the military.

5. ***Smoking cessation counseling***—All enrolled patients have the opportunity to receive multi-call telephone counseling. To meet study Aim 3 described above, we randomize participants to receive this counseling from a VA counselor or from their state quitline.

*VA counseling*: For patients randomized to receive counseling from a VA counselor, they are assigned a VA counselor trained to work with smokers who have a mental health diagnosis. The counselor provides structured counseling using a protocol that we created specifically for this study. The protocol includes proactive counseling and relapse-sensitive scheduling. The content is based on motivational interviewing (MI) and problem solving therapy and addresses both behavioral and cognitive issues, including motivation, self-efficacy, difficult situations, comorbid mental health symptoms, coping strategies, medication usage, and relapse prevention. The counseling includes planning (pre-quit) and follow-up (post-quit) sessions.

*Planning sessions*: The planning sessions last approximately 30–60 minutes and help patients develop an individualized quit plan. Content areas include smoking and quitting history, motivation, environmental factors, planning, proper use of quitting aids, and setting a quit date. Patients receive one to six planning sessions.

*Follow-up sessions*: Patients receive follow-up calls at 0, 1, 3, 7, 14, 21, and 30 days after their quit date. The calls are intended to prevent relapse and to help those who relapse or slip resume quitting. Follow-up calls, which last 10–15 minutes, also use a manual for consistency and fidelity monitoring. Participants are able to call the program staff in between scheduled calls if desired.

*State quitline counseling*: For patients assigned to state quitline counseling, a research assistant initiates a “warm transfer” of the patient to their quitline via a three-way call to start the counseling process. After the initial three-way call, study personnel are not involved in any aspect of the quitline counseling. The quitlines follow their regular service protocols, which are described in Table 2.

6. ***Follow****-****up***—All patients enrolled in the study receive a call from a research assistant at two and six months. Patients who are abstinent from smoking are congratulated, but these assessment calls do not provide counseling. Patients who have relapsed at six months are asked if they want to go through the program again.

**Table 2:** Overview of state quitline services

**State**	**Typical number of counseling sessions**	**QL-initiated calls**	**Client-initiated calls**	**Typical length of first session (min)**	**Typical length of Follow-up sessions (min)**	**Free medications**
MA	6	Y	Y	45	20	Patch
NH	4-6	Y	Y	45	20	None
NJ	6	Y	Y	30	15	None
NY	1	Y	Y	20	10	Patch, gum
RI	5	Y	Y	45	15	None
VT	2	Y	Y	30	20	Patch, gum, lozenge
CT	5	Y	Y	15	10	Patch, gum, lozenge

*QL-initiated* = quitline proactively calls clients to deliver counseling; client-Initiated = clients call into the quitline to receive counseling.

### Counseling standardization and fidelity

State quitlines have standardized protocols and well-trained counselors providing counseling. For the VA counseling arm, the telephone counselors undergo an initial training consisting of two-to-four hours of MI training with a clinical psychologist and 20 hours of training on the study’s clinical protocols. This training includes role-plays with each other and with the study’s project director. After the project director has determined that a counselor has met the role-play training objectives, the counselor advances to complete a series of standardized patient (SP) encounters. The SP encounters involve calling an actor trained to portray a smoker enrolled in the study and completing planning and follow-up counseling sessions per study protocol. The SP encounters are audiotaped and reviewed by a clinical psychologist and the study’s project director for adherence to protocols and counseling techniques. To ensure TeleQuitMH counseling standardization and fidelity after study implementation, the counselors complete clinical documentation using a template that allows them to indicate which protocol objectives were covered during each call and the timing of each call. The study’s project director completes regular audits of the counselor call documentation to ensure adherence to the protocol. The counselors also attend weekly group supervision meetings with a clinical psychologist, during which time the counselors’ active cases are discussed.

### Implementation strategies

We are using a series of implementation strategies at each site to improve the implementation process and increase the uptake and sustainability of the TeleQuitMH program. We used the Promoting Action on Research Implementation in Health Services (PARiHS) [20] and the Predisposing, Reinforcing, and Enabling Constructs in Educational Diagnosis and Evaluation (PRECEDE) [21] theoretical frameworks and evidence from the implementation science literature to guide our choice of implementation strategies. The PARiHS framework suggests that the success of implementing an evidence-based program into practice is determined by three factors: the *evidence* supporting the program, the implementation *context*, and the process of implementation *facilitation*[20]. The PRECEDE framework suggests that changing provider behavior to use a new intervention is enhanced by strategies that *predispose*, *enable*, and *reinforce* behavior change [21]. Based on these frameworks, we chose four primary implementation strategies as described below: blended facilitation, provider training, informatics support, and provider feedback.

#### Strategy 1: blended facilitation

Based on the PARiHS model, we use a combination of external and internal facilitation to implement the TeleQuitMH program. External facilitation is conducted by the study’s principal investigator (PI) and project director, who meet with local stakeholders at each site to recommend methods for successfully implementing the care coordination program based on the methods found to be successful in the primary care-based projects. Internal facilitation is accomplished by each site’s PI, who is responsible for serving as a local champion for the project or identifying someone at their site who would be appropriate to serve as a local champion. In addition, as described in the medication coordination section above, we form a Clinical Advisory Committee at each site to guide local implementation of the TeleQuitMH program. The committees include local representatives from the Departments of Psychology, Psychiatry, Social Work, Smoking Cessation, and Pharmacy. In addition to the committees’ responsibilities in deciding the medication coordination structure for their site, the committees provide guidance on how to inform local providers of TeleQuitMH, provide input on any foreseeable barriers to implementation and provider adoption, and decide how the TeleQuitMH program should work with or complement any existing smoking cessation programs. The committees meet regularly before site TeleQuitMH implementation and as-needed after implementation.

#### Strategy 2: provider training

Based on the PRECEDE model, to predispose providers to utilize the TeleQuitMH program, study PIs present the program to mental health providers at each site during regular staff meetings and via email newsletters. The presentations focus on presenting the evidence behind the program, including its effectiveness in VA primary care (including among mental health patients referred through primary care). The presentations also include a discussion on how the TeleQuitMH program functions within the context of each site’s existing mental health structure and smoking cessation programs.

#### Strategy 3: informatics support

The PRECEDE model posits that enabling interventions simplify the implementation of a recommended practice for providers. To enable the use of TeleQuitMH, we create a local electronic consult for the program that allows providers to refer patients with three-to-four extra clicks as they complete a regular clinical progress note in the EMR system. The consult is also attached to the tobacco clinical reminder and mental health assessment templates in the EMR system, with which providers are already familiar. For providers who do not have clinical privileges to send consults, the study’s project director also accepts referrals via secure email.

#### Strategy 4: provider feedback

To reinforce use of the TeleQuitMH program, we use two strategies. First, we document all patient outreach and counseling sessions with a clinical progress note that mental health providers can read in the EMR system. We also add referring providers as “additional signers” to key TeleQuitMH progress notes, such as notes documenting their patients’ enrollment in the program, their patients’ quit plans, and their patients’ completion of counseling. This allows providers to see their patients’ progress toward quitting smoking and to view the TeleQuitMH program as a regular source of care at their VA facility. Second, we send a regular TeleQuitMH electronic newsletter from a local opinion leader to all mental health providers at each site. The newsletters include instructions for referral and detail site-specific progress with the intervention.

### Patient measures

Table 3 provides details on planned patient measures and timing of administration. Participants complete a survey at baseline, two months, and six months after enrollment. Participants complete the surveys over the telephone with research assistants. We make 10 attempts over one month to reach participants to complete the surveys. The surveys include a demographics questionnaire designed by the study team; a smoking behavior and history questionnaire designed by the study team; the BASIS-24 scale measuring recent mental health functioning [22]; an impulsivity scale [23]; a functional health literacy scale [24]; measures of readiness to quit, motivation, self-efficacy, and attitudes about smoking developed by the study team; and satisfaction with treatment received from the TeleQuitMH program.

**Table 3:** Patient measures and assessment schedule

**Measures**	**BL**	**2m**	**6m**
Sociodemographics	X		
Current and historical smoking behavior	X	X	X
Readiness to quit	X	X	X
Quitting self-efficacy and motivation	X	X	X
Medication assessment: Current use, contraindications to NRT or Bupropion	X		
Use of prior smoking cessation treatment	X	X	X
Nicotine dependence	X	X	X
Alcohol and substance abuse	X	X	X
Smoking environment: Social support for quitting, household smokers, household smoking rules, employer smoker rules	X		
Attitudes about smoking	X		
Mental health symptoms (BASIS-24)	X	X	X
Health literacy	X		
Impulsivity	X		
Patient satisfaction with the program		X	X
Patient assessment of treatment fidelity/counseling content		X	X

*BL* = baseline; *2m* = two months after enrollment; *6m* = six months after enrollment.

### Formative/implementation evaluation

The study includes a formative evaluation to help us modify the care coordination program and implementation strategies during our study period, to enhance the interpretation of summative data, and to better inform future implementation efforts. Table 4 provides details on formative evaluation activities and measures. We used the Stetler et al. [25] model to guide the development of our formative evaluation methods. This model identifies four stages of formative evaluation for implementation research:

1. ***Developmental phase****—*Based on our experience with the primary care-based projects, we anticipate that it will take six months to implement the TeleQuitMH program at a site. This time includes identifying a local champion, obtaining Institutional Review Board approval, implementing the TeleQuitMH program’s main components, and informing providers. During this time we will conduct mental health provider surveys and interviews with site stakeholders to measure 1) the level of organizational readiness of the participating clinics both in general and specific to the intervention, 2) perceptions of the evidence supporting smoking cessation telephone counseling and smoking cessation medications, 3) perceptions regarding the feasibility and utility of the planned intervention, and 4) perceptions of anticipated barriers or facilitators to the successful implementation of the intervention. We will also assess the extent to which each site’s local champion and Clinical Advisory Committee are interested in, and actually perform, their project responsibilities, assess the structure and processes of the site’s state quitline operations (e.g., hours of operation), and assess the type and intensity of local smoking cessation services already available to mental health patients. We will formulate modifications to the TeleQuitMH program structure and our provider outreach/marketing efforts to address findings from these activities.

2. ***Implementation****/****progress****-****focused phase****—*For the purpose of our evaluation, we will combine Stetler et al.’s *implementation* and *progress-focused* phases. After implementing the TeleQuitMH program at a site, we assess several patient and provider variables on a regular basis, including the number of patient referrals, the percentage and types of providers who are referring patients, the percent of patients enrolling and engaging in treatment, and patient satisfaction with the program. We also conduct a second provider survey during this phase evaluating reasons for a provider’s referring or not referring patients to the program, provider satisfaction with various aspects of the program, and what, if any, recommendations providers have for changes to the program that they feel would improve its effectiveness. We also assess referral trends in relation to our provider outreach activities in order to modify or target future outreach efforts. Last, we continue to assess the impact of each site’s TeleQuitMH components (e.g., prescribing structure, referral process) on implementation fidelity, referral rates and patient uptake, treatment fidelity by the VA counselors, and the presence and quality of facilitation provided by the local champions and Clinical Advisory Committees.

3. ***Interpretation****/****sustainability****-****focused phase****—*Following the intervention period at each site, we collect two additional measures: an organizational survey of mental health programs, which assesses each site’s mental Health clinic structure, size, staffing, environment, and resources [26]; and mental health unit performance rates on the mental health treatment and tobacco use clinical reminders required by the VA [27]. We use these data and the formative evaluation data collected during the previous two phases to describe the local implementation context, to formulate interpretations of primary outcomes data, and to create recommendations for future implementation efforts of the telephone care coordination program.

**Table 4:** Formative evaluation (FE) measures and evaluation schedule

**FE Measure/ activity**	**Measure/activity description**	**Develop-mental**	**Impl./ progress focused**	**Interpret./ sustainability focused**
**Reach/Implementation Analysis**				
Referral rates	Number of patients referred on weekly and monthly basis.		**X**	**X**
Treatment uptake	Percent of referred patients enrolling in treatment, engaging in treatment, and completing treatment.		**X**	**X**
Exposure to TeleQuitMH	Percent of MH patients for whom tobacco use screening was completed.			**X**
**Patient Process Data**				
Enrollment Rates	Rates of enrollment to TeleQuit MH and State quitlines		**X**	**X**
TeleQuitMH Satisfaction	Self-reported satisfaction with TeleQuitMH structure and treatment (including VA versus quitline satisfaction) ; assessed 2m and 6m after enrolling		**X**	**X**
**Provider Process Data**				
Provider Survey	Perceptions of program marketing; how likely they will be to use the program; barriers to implementation; suggestions for improvement; provider use of the program; program satisfaction; perceived effectiveness of activities to facilitate implementation; impact of the program on their patients/client.	**X**	**X**	**X**
Stakeholder Interviews	Perceptions of program marketing; how likely they will be to use the program; barriers to implementation; suggestions for improvement; provider use of the program; likes/dislikes about the program; impact of the program on their patients/clients.	**X**	**X**	**X**
Referral rates/patterns	Percent of providers referring; Number of referrals from each provider; Rates by provider type		**X**	**X**
**MH Unit Data on Implementation Context**				
VA Survey of MH Programs[28]	Clinic’s structure, size, staffing, environment, and resources for meeting MH and tobacco cessation treatment goals.			**X**
MH Unit Performance Data[29]	Clinic-level performance on VA MH and tobacco performance measures			**X**
**Implementation Facilitation - Structure**				
Site Champion	Presence of a site champion, level of interest in performing site champion duties, position at the VA, role on the project	**X**	**X**	**X**
Site Clinical Advisory Committee	Presence of a site Clinical Advisory Committee, level of interest in performing Committee duties, Committee members positions at the VA & roles on the project	**X**	**X**	**X**
Site smoking cessation services	Presence, types, and structure of site smoking cessation clinic and other local smoking cessation services	**X**		**X**
TeleQuitMH Structure	Structure of the TeleQuitMH program at each site, including design and appearance of the tobacco cessation clinical reminder, prescribing structure, and referral mechanisms (e.g., consult design, email referral option)	**X**	**X**	**X**
State quitline Structure/Issues	Structure of quitline services; issues/problems identified in connecting TeleQuitMH patients to quitline services.	**X**	**X**	**X**
IRB Issues	Time from first submission to receiving IRB approval; type of submission required (full board review, expedited, exempt); site-specific requirements that may affect TeleQuitMH implementation and adoption (e.g., detailed consent process)	**X**		**X**
**Implementation Facilitation - Activities**				
Marketing Efforts	Provider outreach efforts, academic detailing, provider feedback.		**X**	**X**
Treatment Fidelity	Extent to which the TeleQuitMH staff are providing the telephone counseling, medication coordination and warm-transfer to the state quitlines as intended.		**X**	**X**

*MH* = mental health.

### Summative analyses

We are using the RE-AIM framework to guide summative data analyses [28]. The RE-AIM framework suggests that it is crucial for a system-level intervention to consider the following: *Reach*—the proportion of patients reached by the intervention; *Effectiveness*—the effectiveness of the program at meeting clinical goals; *Adoption*—the proportion of providers and patients that used the intervention; *Implementation*—the extent the intervention was implemented; and *Maintenance*—whether the intervention was maintained over time.

#### Reach

To assess the reach of the intervention, we will determine the proportion of mental health patients who were exposed to the TeleQuitMH program. To do this, we will first use the EMR system to identify all smokers at participating facilities who had a mental health visit during the intervention period. This group—all smokers with a mental health visit—will be our denominator. If the TeleQuitMH-adapted tobacco use clinical reminder appeared for the patient’s mental health care provider, we will consider that patient to have been exposed to TeleQuitMH. *Reach* will be calculated as the number of smokers exposed to TeleQuitMH divided by the total number of smokers with a mental health clinic visit.

#### Effectiveness

Study Aims 2 and 3 examine the clinical outcomes of the TeleQuitMH program. For Aim 2 (*Determine the effectiveness of telephone counseling in promoting long-term abstinence from smoking among mental health patients*) there will be no control group, as these data will only be collected on patients who enroll in TeleQuitMH. We will calculate the percentage of participants with 30-day point prevalence abstinence at two and six months. We will also examine which baseline characteristics (e.g. age, race/ethnicity, self-efficacy, nicotine addiction, mental health diagnosis, symptom severity) are associated with abstinence, and we will use multivariable logistic regression to determine those characteristics that are independently associated with abstinence. For Aim 3 (*Compare the effectiveness of telephone counseling delivered by VA staff with that delivered by state quitlines*) we will calculate the 30-day point prevalence abstinence rates at two and six months separately for the two treatment arms and compare these rates using logistic regression, adjusting for covariables.

We based our power calculation on an ability to detect a difference in six-month abstinence between our two randomized groups. We expect the six-month abstinence rate will be 15% in the quitline arm and 20% in the VA arm. With an anticipated 1200 participants in each treatment arm, we will have >80% power to detect this 5% difference in abstinence rates between the two arms at the 0.05 significance level.

#### Adoption

We will calculate program adoption by providers and patients across all sites and for each site separately. For this study, *adoption by a provider* will be defined as referring one or more patients to the TeleQuitMH program; i.e., the provider used our provider-level intervention of adapting the EMR to enable referrals. To calculate the program adoption by providers, we will generate a list of all mental health providers at each site using administrative records and a list of all mental health providers who made at least one referral using study data. We will then divide the number of providers who made at least one referral by the total number of providers. *Adoption by a patient* will be defined as enrolling in treatment. We will have a list of all patients referred to TeleQuitMH and the list of patients who enroll in treatment. We will divide the number of patients who enroll in treatment by the total number of patients referred to the program.

#### Implementation and maintenance

We will assess the program *implementation* at each site by examining the activities occurring at each site. There are eight main implementation activities that need to be completed to fully implement the TeleQuitMH program at a site (Table 5). We will score each site on the completion, fidelity, and intensity of each of these eight activities using anchors developed by the study team. We will assess program *maintenance* by examining monthly referral rates to examine time trends in referrals and whether patients and providers continue to use the program for the full intervention period.

**Table 5:** Major TeleQuitMH implementation activities at each site


1. Designation of a local champion for the program	
2. Creation of a site Clinical Advisory Committee	
3. Creation of site prescribing structure	
4. Modification of EMR system to enable referrals and local documentation of treatment delivery	
5. Permission and process to make warm-transfers of patients to the state quitline	
6. Program marketing to providers	
7. Provider referrals	
8. Delivery of smoking cessation counseling and smoking cessation medications to enrolled patients.	

### Formative evaluation analyses

#### Developmental phase

Baseline provider survey and stakeholder interview data on attitudes toward smoking cessation and perceived barriers to telephone smoking cessation will be used to refine the implementation of the intervention. Documentation of the core structural components of the implementation at each site (e.g., prescribing structure, pre-existing smoking cessation services, characteristics of local champion) will be used to ensure the best possible implementation given site circumstances.

#### Implementation/progress focuses

Referral rates, treatment uptake and exposure at the patient level, and responses to TeleQuitMH at the provider level (collected via surveys, stakeholder interviews, and referral rates/patterns), will be continuously monitored to track program progress and identify sites that may need additional activities and efforts focused on facilitating implementation. Information on the structure of the implementation as it unfolds will be reviewed at regular staff meetings and linked with initial patient (enrollment rates, satisfaction) and provider process data (referral rates and patterns) to determine next steps for implementation including directed outreach/marketing; changes in structure, etc.

#### Interpretation/sustainability phase

After the intervention period has ended at each site, we will use the *interpretation/sustainability phase* of our formative evaluation to interpret primary outcomes, using qualitative pattern-matching approaches to explore how the implementation activities and structure of the implementation (including treatment fidelity) may help explain variations in outcomes at the site level. Similarly, data on the implementation context will be analyzed to determine variation across sites and whether that variation is associated with both the nature of the implementation and the outcomes achieved. Formative evaluation data will be analyzed longitudinally to determine if changes in implementation are associated with changes in process variables (e.g., reach, uptake). In terms of sustainability, analyses will focus on identifying the site and implementation features and characteristics that facilitated and hampered effectiveness. Finally, descriptions of the degree to which core elements of TeleQuitMH were integrated into systems of care at each site will be used to characterize the sustainability of TeleQuitMH and to identify the local conditions which facilitate replication and dissemination.

### Economic evaluation

It is not within the scope of this project to conduct a cost-effectiveness analysis. For this project, we will assess the costs involved with the TeleQuitMH program, including staff costs (salary and fringe benefits), cost of smoking cessation medications, and VA overhead. We will use data from the VA’s Decision Support System for the costs of provider time and smoking cessation medications. We will calculate the cost associated with counseling delivery using study administrative records for the VA counseling arm and quitline program estimates for the quitline arm. We will use VA rental space agreements at our primary site to estimate the cost of VA overhead associated with the program.

## Discussion

It can take 17 years for new clinical interventions to make their way through the efficacy-effectiveness-implementation research pipeline and become usual clinical care that improves patient outcomes in routine practice settings [29]. Hybrid effectiveness-implementation study designs offer the potential to greatly shorten this translation time [19]. This study utilizes an innovative hybrid design to concurrently study the clinical outcomes and implementation into routine care of a smoking cessation telephone care coordination program for mental health patients in six VA facilities. The study will study the clinical effectiveness of the care coordination program and compare the effectivness of two models for connecting mental health patients to telephone smoking cessation counseling (a tailored VA-staffed program versus a warm-transfer of patients to their state quitline), while producing important data about the acceptability and feasibility of telephone-based smoking cessation treatment among mental health providers and patients. Formative evaluation data collected during study start-up and regularly throughout the intervention period will help future tobacco control efforts in mental health treatment settings by identifying barriers to and facilitators of successful treatment program implementation.

The study will have several limitations. First, we will rely on self-reported abstience, which may produce over-estimates of short- and long-term quit rates among participants. Second, there is considerable variability in services provided by the quitlines, and we will not have sufficient power to conduct Aim 3 analyses separately for each state. Third, we randomize at the patient-level and will not be able to determine the effectiveness of our implementation strategies. However, researchers can use the qualitative and nonrandomized implementation outcomes data collected during the trial to design future group-randomized studies evaluating the effectiveness of different stratagies for implementing tobacco cessation treatment programs in mental health clinics. Lastly, we will not have data on the number or duration of counseling sessions provided to participants connected to the quitlines for counseling, so we will be unable to account for treatment intensity in our Aim 3 analyses.

## Competing interests

The authors declare that they have no competing interests.

## Author contributions

SES, DS, and ER conceptualized the study and obtained funding. ER is leading the team in implementing the study. ER, SF, and DK designed and implemented the telephone counseling protocols for the VA counseling arm. HJH and CG designed the formative evaluation, and CG is implementing the formative evaluation and will conduct all formative evaluation analyses. BE designed and will perform cost-related analyses. All authors contributed significantly to the design and implementation of methods for collecting and analyzing clinical effectivness data. All authors contributed to the preparation of the manuscript.
